# Cognitive Accessibility and Support in Special Education

**DOI:** 10.3390/s21144871

**Published:** 2021-07-16

**Authors:** Miguel A. Guillomía, José Ignacio Artigas, Jorge L. Falcó

**Affiliations:** Department of Ingeniería Electrónica y Comunicaciones, Universidad de Zaragoza, María de Luna 1, 50018 Zaragoza, Spain; magmcm@yahoo.es (M.A.G.); jiartigas@unizar.es (J.I.A.)

**Keywords:** ambient assisted living, cognitive support, social training, special education, assistive technology, personal autonomy, human–machine interaction

## Abstract

This work describes an assistive technology development for cognitive support and training to be used by children of special education schools in Spain. Design is based on and guided by cognitive support findings coming from a long-term collaboration of a team of engineers (University of Zaragoza) and special education teachers (Alborada Special Education School, Zaragoza). The description starts by providing a structure of such findings in five cognitive-social areas (interface usability, virtual representations understanding, time orientation, self-awareness, and social interaction). Design requirements are extracted by applying those findings to four support services (home control, time orientation, behavior contention, and context anticipation). Technological system description follows, together with the degree of implementation and testing for each service. A major result is the benefit of using a services interface with the same structure and appearance as the alternative and augmentative communication system that children are already acquainted with. Based on regulatory conditions, the needed flexibility, and reduced available budgets, this support platform is built on mainstream technology and low-cost single-board computers with standard databases and free software packages. Results show evidence of benefit in children’s cognitive and social performance, in addition to offering a technological tool for deeper educational research.

## 1. Introduction

This paper describes a platform designed and developed to provide cognitive support through assistive technology services to children in special education schools. Technologically, it relates with ambient intelligence and ambient assisted living in which services are integrated. Regarding human factors, it incorporates multimedia and immersive technologies with a user interface based on their current Alternative and Augmentative Communication (AAC) system. The paper starts with the rationale of cognitive support features, which are gathered from previous experiences and used to guide the current design, resulting in user requirements for the platform and services. Then it focuses on the technological options and design keys of the platform considered to describe the technological structure implemented. Then each service is described both at the functional and technological level. Results of its implementation and evaluation (when available) are included.

Ambient Assisted Living (AAL) is a specific Ambient Intelligence (AMI) paradigm that focuses on human functions support for people with special needs. The AMI vision described by Weiser [1] started the AMI paradigm. AAL appeared for the first time in the European Framework Program for research funding. Cognitive functions are probably the most difficult to give support to, due to their complexity compared to sensorial or motor abilities, and also due to the need for some cognitive ability to access the support system. This work focuses on supporting cognitive functions aimed to increase the autonomy of children of special education, including those that affect their social skills. It is framed in long-term cooperation [2,3] of an engineering school and a special education school in Zaragoza (Spain): special needs reality is shown to engineering students as part of their education and some of their projects are oriented to give solutions to children’s necessities.

A key factor of this work is introducing their AAC system as guidance for the interface for all services. It is a pictogram-based communication editor called TICO [4]. TICO is a communication panel editor and interpreter which is solidly established in this field. It is mainly based on ARASAAC [5] pictograms which offer a large set of free pictograms with an important impact in AAC communities. Services also use multimedia immersive environments and elements of a sensory stimulation room.

### 1.1. Developed Services Short Description

Four services are used to try and implement the described cognitive and social support and training platform. Their introduction at this stage of the paper responds to the need to center the state of the art survey, for it will study keys of cognitive aspects to attend and previous work done in similar services in AAL.

#### 1.1.1. Home Control

Cognitive accessibility is added to a home control system that operates on switches, shutters, multimedia equipment, lights, etc. It uses mainstream hardware. A new interface based on TICO was developed to improve its cognitive accessibility, having very good results. TICO is considered a previous cognitive basis of participant children for they are trained and acquainted with it. Home control navigation structure follows TICO’s nesting semantic structures: TICO uses it to find the aimed pictogram; home control uses it to find the room, devices and function to be triggered. Aesthetics also are copied in the home control interface.

Home control service has been installed at school for two years. It has proved to have enhanced accessibility and usability. It has also proved useful as a training tool for virtual representation and cause-effect comprehension [6].

#### 1.1.2. Time Orientation (TO)

This service was considered highly relevant for children’s autonomy in 2017 by the psychology department of the special education school. It is a major cognitive training challenge due to the abstraction level involved. It uses a line of luminous elements. It represents the time passing concept by turning them off sequentially, so it also provides for a time location display. Pictograms with agenda events are displayed or placed beside the luminous elements. Color codes differentiate past, current, and future events. Class agenda is them showed with time location and time duration for each event in the day. Individual events are identified with the picture of the pupil involved. Acoustic or luminous information is used to anticipate coming tasks, which is thought to help reduce anxiety associated with changes, especially in children with autism and hyperactivity disorders.

Its implementation in more than ten classrooms for several months has given measured improvements in autonomy related functions [7,8]. Advances from former prototypes have included direct integration with the educational service platform and TICO-like interfaces, wider configuration options, direct import of programmed agendas from TICO panels, multiuser dynamics, cognitive access, and social interaction features.

#### 1.1.3. Behavior Contention

Children in special education schools frequently show insufficient emotional contention capacity, so an important autonomy drawback is the arousal of disruptive behaviors which may ruin their individual performance and their social participation. Emotional regulation is critical in disruptive behavior contention. Teachers present the child with a sequence of behavior rewards, such as staying still or silent for some minutes or stopping an aggressive impulse. As contention is set in a time interval, time passing progress display is good feedback to support children’s self-management, which also helps raise awareness of time perception and of their internal emotional status.

#### 1.1.4. Context Anticipation

This service tries to immerse children in different contexts to train specific skills. Multimedia immersion also seeks to influence the activation rhythm or mood of the children. It uses sensory stimulation room elements and experience, and multimedia projection features in surrounding screens to provide relaxing or activating scenarios. It uses role-playing dynamics to expose children to situations to train their behavior and emotional management.

As an example, children in higher autonomy classes used to go to a nearby cafeteria, ask for a soda, pay for it and have it there with their classmates. This activity is found to produce stress on them. Context anticipation would project on surrounding screen images resembling the cafeteria scenario and each participant would acquire a role to play, in order to train children to overcome potential emotional or mental blockages. Specific challenging moments can be softened by slowing down the rhythm of role playing, and by adjusting scenario parameters as done in sensory stimulation rooms. One such moment is when children have to check the change received after payment.

### 1.2. Innovations and Previous Works

This work originated by focusing on cognitive accessibility for environment control for children who failed in using standard programs. The incorporation of an AAC language into home control was published in [6]. The aim was to solve the burdens of access, understanding, and usability. It highlighted important cognitive issues that have later been integrated into the AAL platform, as the association between virtual and real representations or the parallelism between nesting navigation fields and grammatical structures for TICO.

Innovation work from that publication includes the extraction of cognitive features to incorporate as horizontal cognitive support and also the incorporation of a multiuser mode.

A second previous work that incorporated TICO and multimedia was the time orientation service, whose interface was designed as a combination of their class agenda and their pictogram communication interface. Its aim was to train in time management at different levels. It was published as an independent service in a congress [7] and as a paper in [8]. Its goals were increasing temporal awareness, the ability to address changes minimizing associated stress (especially in autism), and agenda management for the most advanced pupils in the “transition to daily life” educational program.

From that point, the temporal orientation service has been redesigned to include:

1. Cognitive accessibility: adaptation of time service to AAC scheme, with the identification of new needed pictograms related to time. Service configuration may be done directly in the TICO panel editor and imported then into the TO service.
2. Contextual time information: service information structure was merged with others into a common database to allow authorized services to use agenda information.
3. Multiuser configuration: it has been added to integrate this service with the social dimension aimed and provide for group agenda activities of planning and follow up.

A third step was the use of the time orientation service as a behavior contention support tool. It was used by teachers to give children feedback on the time left to obtain a reward while keeping a behavior containment. Following the success of this strategy to manage certain disruptive behaviors, a new service was designed. It showed in pictograms the behavior sequence to which the reward was conditioned, and displayed time left with lighted dots, as time orientation service does.

A fourth previous work to mention relates to immersive environments and sensory stimulation rooms. At school, they have had success exporting some sensory room ideas to a multiuser-space with surround video projection, sound, and other elements such as wind, humidity, temperature, odor, which were used in physical education class: “the cube” [9]. This previous work constitutes an experiential reference for the immersive capacity of video display and its effect on children’s motivation and mood in this special education school.

Behavior contention trials together with the potential of affecting children’s mood by immersive environments, also raised the importance of training in emotion management to improve children’s social capacities. Many children’s emotional and behavioral burdens show up in group dynamics by contrast with individual work. Children could benefit from training in sustainable social behaviors and emotion re-conduction.

This social dimension was then extended to all services that were re-developed to be used not only individually, but also in group-class education dynamics. Multiuser feature applied to immersive environments as sensory stimulation rooms, brought up a new service to facilitate role-playing in simulated environments, which became our fourth service: context anticipation. It became straightforward to incorporate our previous work with technology for the multisensory stimulation room into the immersive role-playing scenario. Context anticipation service originated to train emotional, comprehension, or behavioral difficulties in specific contexts, using immersive multimedia technologies and sensory room elements.

Services were re-designed to support cognitive parameters identified, class (-group) activities, and social training. The system gives support to individual participation in group activities by allowing several simultaneous interfaces with various cognitive complexity levels. To achieve this, a new software structure has been implemented.

## 2. State of Research

The authors have not found similar works to the one presented of a technological system aimed specifically for global cognitive support based on known communication schemes. The following survey describes works that provide experiences and insight into relevant aspects for cognitive support and accessibility. Those works are consequently classified by such relevant aspects, such as usability, human-machine interfaces, using information technologies for social skills training, emotional management, self-awareness, and time orientation.

This study also includes papers describing similar specific support services, as home control and temporal orientation. There is a large set of works about technological platforms that integrate several AAL services, most of them with no specific focus on cognition aspects, yet mainly centered on technological advances, interoperability, and strategies of information merging.

### 2.1. Usability, Cognitive Accessibility, Virtual Representations, and Interfaces

The literature points to ICT as a valid instrument for cognitive accessibility, personal autonomy, and natural interaction, showing specific benefits in education [10]. ICT provides for simultaneous and combined use of files and languages in different formats as sound, static and dynamic images, symbols, and other representations, which is the key to its capacity to enhance cognitive accessibility [11]. ICT’s large potential in learning is closely related to its possibilities to display, process, transmit and share data, offering students almost unlimited access to information [12]. Anyhow, cognitive tools and educative practices based on ICT are rare and incipient [12].

Technology use for special needs is widely supported [13]. Its acceptance and usability, though, are related to the combination of human and technical aspects of a design. Acceptance of technology is influenced by its accessibility, usability, and functionality. Only the last is reached if development focus is set in technology alone [14]: an expected critical issue in the forthcoming years is technology acceptance. Acceptance shows high variability, so the importance of integrating diverse user groups into the design and evaluation process of AAL technologies is stressed [15]. Of course, there is no absolute separating line between technology and user experience, as shown in [16] when considering seamless and coherent user experience based on seamless technological solutions. Looking at AAL from a functional or user perspective, some equilibrium issues arise that affect ethics and consequently will affect acceptance, as caregiver supervision vs privacy, over-reliant AAL solutions, and dependency vs peace of mind [17].

Key concepts for cognitive accessibility are studied in [18]: information processing, understanding, easy reading, signaling, which are studied in two support applications: communication and indoors orientation. Additionally, it highlights that cognitive and accessibility benefits all populations, not just people with special needs. Some web design regulations provide for specific key aspects in cognitive accessibility, as found in [19,20,21].

Virtual reality understanding opens a world of resources for people with special needs in accessing ICT, as in indoors orientation applications [22]. Tangible interaction is another interaction way for ICT interfaces where cognitive support is relevant, as tested for special education in [23]. All these findings suggest a revision of ICT issues in education for teachers at university, which are found insufficient in [24].

Interfaces for Intellectual Disability (ID) in AAL are often based on pictograms, which offer a communication way by relating concepts [25]. Amount and contextualization of information are especially focused on specific interfaces as in Activepal [26]. Context information and user information facilitate adaptable interfaces and modeling interaction to extract and share information among applications or services, which can be resumed in methodology guides [27].

Some works anticipate that user communication methods are a good strategy to obtain better cognitive accessibility by introducing individualized and adaptive interfaces [27,28].

Cognitive accessibility is a burden to overcome to obtain social participation. Interface design should look from this standpoint into its appearance, usability, and content [29]. Social integration and children’s motivation benefit from ICT [30]. It also benefits integration in normalized contexts such as national education plans for special needs, as described in [31] based on real experiences with children with autism spectrum. A classification of available ICT resources in autism can be found in [32]. The lack of accessibility to ICT produces new discrimination patterns in our society, the so-called “info-exclusion”, increasing the disadvantage of other exclusion patterns as disabilities [33].

### 2.2. Social Interaction and Training with ICT Support

Motivations to accept and use ICT are a challenge for ICT developers [34]. The work in [35] identifies six categories of incentives and states “social connectedness” as the most important incentive together with “health and safety”, highlighting this way the importance of social participation for children and their social environment.

One aspect of special education is the inclusion of children in normalized environments. Some works point out this tendency as the main role to play for ICT in children with special needs to support their autonomy, cognitive and social development [36]. Some authors indicate that benefits in the use of ICT for learning are conditioned by the perspective taken, and they suggest it should be based on experiences of social interaction, active participation, and support in complex environments, again highlighting social dimension as a basic goal to achieve when looking for personal autonomy [37]. Together with daily life activities performance, social skills, and social and behavioral development are to be interrelated to support the academic performance of children with intellectual disabilities [38].

Specifically, social relations are the main objective in interventions in mental disability or cognitive disorders to improve their performance in life as studied in [39] through the revision of 137 papers published between 2007 and 2016 in different databases. Such special social difficulties often come as a defensive attitude, problem-based strategies, and frequent anguish crises, provoking an increase in stress levels.

Several experiences insist on the efficiency of those approaches. Children with mental disabilities are trained in listening, respecting social distances, interacting in games, respecting each other, and asking for issues in an educated way [40]. The work in [41] shows different techniques used to develop social skills based on learning theories from different paradigms, studying comparative advantages and disadvantages.

### 2.3. Keys in Social Interaction: Communication and Emotional Management. Sensory Room Elements

Social interaction requires from children some skills in communication, social codes (referred to as social distances and times), behavior contention, and emotion management, as shown by literature.

Communication has evolved from a direct presence in the real world into the virtual world, bringing social interaction into this new dimension, where technological support is natural [42]. This work shows experiences in which training young people with intellectual disabilities in virtual social interaction results in success in boosting their social skills.

The communication process can be understood as a compound of emission, transmission, and reception: in our target population, a critical part is the understanding of the information received, which [43] signals as the main burden for the social life of children with cognitive disability. Training in sequence and significance of received information improves normalized social interaction. Following this idea, socialization uses normalized codes which in the end are cognitive patterns that children need for their social integration. Training children in such codes and patterns are found to be effective ways to promote better social participation for children with ID or disorders of the autistic spectrum [44].

Emotional management, as well as social skills, are basic in the social interaction of children with ID [45]. Cognitive development and language skills have a large influence on emotional management performance. Other social skills as cooperation, inclusion, and control of disruptive behaviors are basic elements for social participation inside the classes. Emotional intelligence’ theoretical basis can be applied to children training in this population, having a large impact on social skills. Communication, interaction, and behavior are some of the points to improve at early ages so they can work later in self-concept and social skills. This also serves as a prevention mechanism for other risk situations such as conflicts, aggressiveness, uncontained impulses, and behavior problems among others [46]. Adaptive behavior requires emotional control and is proposed as a normalizing tool for children with ID in [47]. Results in training increase the self-esteem of participants, an aspect of self-awareness.

Literature also includes sensory room technology as suitable for social conduct training. In [48], experiences with free technology used in sensory rooms resulted in an improvement of the social behavior of participants. The work in [49] also highlights the efficiency of those environments to train communication codes, time and space perception. It also concludes their later positive influence on children’s social skills. ICT plays an important role in sensory stimulation and skills development for social integration [50,51]. Education may see ICT as a tool to be combined with educative practices to offer multisensorial, attractive, and very motivational experiences [52].

### 2.4. Home Control and AAL Platforms

Home control service design was built in the same hardware in which the platform is currently deployed. Previous works in European AAL projects for seniors provided our team with an idea of technological trends as far as platforms in AAL. A review of the AAL platform and interfaces is shown in [25], compiling information about some widely-known AAL platforms as Soprano, Universaal, MonAmi (OSGI), i2home, and others. Also [25] highlights the need to address cognitive issues as how the elderly absorb different pieces of information and how this may lead to differences in product acceptance by the markets.

Technological challenges for AAL platforms included interfaces and interoperability across various technology standards, and among the different services, regarding internal information sharing. An efficient way to provide for data sharing and interoperability is the use of databases [53]. Several works show interoperability solutions, from sensor networks [54] to full operating platforms. There are several efforts to obtain interoperability for technology interfacing [55,56,57,58,59]. Among them, URC implemented a wide technological interaction standardization and set a strategy for interoperability that has come later in several other formats [28].

### 2.5. Time Orientation in Human Development, Personal Autonomy, and Participation

The World Health Organization compiled a study in 2001 which classified all human functions regarding human structures, functionality and social participation, the International Classification of Functionality, Disability and Health (ICF) [60]. Regarding time functions ICF addresses them in two different categories: the human body and participation functions. There are three items in body functions: (1) B1140 describes as body function the “knowledge of day, date, month and year the person is”. (2) B1642 is part of superior human cognitive functions as “management of time by ordering events in a time sequence”. (3) B1802 as “subjective experience of time passing”. As participation functions, it is found item 2306: “capacity of adaptation to time demands”.

These same key functions were further elaborated by Gunnel Janeslatt in [61,62], grading time functions on a scale of difficulty or abstraction: time perception, capacity to locate present time (in the day, month…), sequencing tasks, or events to accomplish a goal, calculating the duration of tasks or events, and planning time including time calculations (duration and sequence) and agenda management. This work resulted in an evaluation tool to assess how well a child performs in any of the previous categories, KaTid, which the authors trained on and plan to use in evaluating our time training intervention.

Time orientation can be seen as a capacity in itself and also as an expression of other human capacities, as time awareness and other underlying human capacities. Literature shows how cognitive disability is related to time orientation and how this may lead, for instance, to dementia assessment [63]. It is also used as a measure of daily tasks efficacy as shown in [64]. Time orientation and habits in focusing time also have influence in other areas in life, as it is having and following vital objectives in life. In [65], this is related to personal awareness and influenced by age and gender.

Habits in focusing on the present, past, or future have an impact on vital positioning in life. Taking the future into account favors sustainable behaviors and attitudes [66]. Focusing on the future also has a positive relation with having longer term goals and being able to follow external regulation strategies [67]. This study with normalized young people in the University of Barcelona shows how such focus on the future helps transform behavior patterns and raise awareness of risk in addictions [67].

Focus pattern in time orientation is also found to be related to personal awareness. Attention at present favors internal awareness, and future and past focusing favors external awareness [68].

As far as time orientation for personal autonomy, the implemented display of time passing comes originally from Arne Svensk, who developed a clock based on the turning off of sequential lighting elements to assist people with ID [68,69,70]. He chose a time unit of 15 min as the optimum for better comprehension and operability of the population he worked with. The quarter-hour clock [69] was derived from his experiences. The quarter-hour dot came as a de-facto standard in technical aids to express time duration [70]. Some later developments can be found in [71].

Time orientation service also has a goal to anticipate changes and reduce associated anxiety. Literature shows devices to “anticipate activities” by cards or pictograms, giving time duration information about activities or the time left for them in standardized dots [72]. As far as software apps available, ref. [73] highlights the convenience of tasks and time passing information combined. The work in [74] shows the efficacy of such technical aids to help processing time for people with cognitive difficulties.

Former studies of our research team used pictograms, images, and objects to describe events and tasks associated with a time display in a similar way as A. Svensk’s [75,76].

## 3. Design Methodology and System Requirements

The target population for this system is children in special education schools. The primary scenario is the school. Children’s homes are a secondary scenario, as families are very much involved and concerned with children’s autonomy and they are an active part of the educational community. This implies that services configuration and events programming needs to be kept simple and adaptable, and with remote maintenance and updates capacities.

The work methodology that special education school demands is user-oriented. The research team starts by identifying specific difficulties and potential support solutions. Its potential impact on the user side is assessed considering how many children would be benefited and how important the effect on a child’s autonomy may be. On the technological side, the assessment considers how straightforward a solution can be as far as available technology and time-to-market (i.e., time-to-trials), considering how much can be achieved with a development action and the need for further research. Of course, its estimated cost is also assessed. Once a specific support is defined as a goal, the team chose a child or a group of children to start working with. This raises the need to have user experiences accompanying the previous research, design, development, and testing.

Due to this experimental requisite, installation is needed almost from the very beginning, so mainstreaming devices are preferred when available. Moreover, using mainstreaming technology makes it easier for quick trials installation and its future implementation regarding safety and standard regulations. Single board computers and technological modules ready to integrate are also good options that offer quick development time and flexibility for continuous adaptation guided by human support research.

This work focuses on applicable conceptual items: once the concepts and tools are clear, it is straightforward to adapt our developments to new technological setups, new platforms, sensors, and communication standards, which are framed in research projects or Ph.D. thesis at university. The work presented in this paper has gone through these stages several times until it was installed at school, tested with a larger number of children, and then offered to the educational community through their continuous education network. Only the time orientation service has no-mainstreaming hardware of its own, which poses new challenges for building, marketing, distribution, and maintenance that imply companies and institutions that are out of the scope of this paper.

This section of the paper describes the work performed to integrate the findings of cognitive and social support into the specific services. Its inputs are the cognitive items to be included. Its outcomes are the requirements for the technological development for each service.

### 3.1. Cognitive and Social Supportive Features

A set of cognitive features has been identified based on previous experiences with children in special education. Many came from observing how the use of their AAC as an interface helped children’s performance. Teachers observed when children behave by automatism and imitation, or when they had the cognitive skills integrated, as virtual representation understanding, time orientation, or self-awareness, identifying those key cognitive factors. Other features were induced by researchers by extrapolating potential benefits, as emotional containment and social interaction.

This work identifies five cognitive areas for focus: (1) virtual representation association, (2) improved cognitive accessibility by using already known cognitive structures as languages, (3) time orientation, (4) inner personal awareness, and (5) social interaction. 

#### 3.1.1. Cognitive Support in Virtual and Conceptual Representations

Association between virtual representation and the elements in the real world is used in home control via pictures or pictograms. Home control training showed that children with good skills in this association advanced quickly in learning operations that could be done over such elements, providing an enhanced association between pictograms and actions.

For children with little previous skill in this area, home control usage revealed their difficulties while also proving to be a good training tool. This success in training was explained by the enhanced relation that the home control system evidenced among virtual elements (images and actions) and their associated real elements through direct cause-effect.

After testing and training with several children, the virtual representation area was decomposed into three items:

1. the semantics of virtual elements
2. functions or operations to be done over such elements
3. cause-effect training.

This cognitive area is found crucial for the target population, for it may open the virtual world to children otherwise apart from it. This may widen their autonomy into occupational activity, social relations, access to practical information, and leisure.

#### 3.1.2. Cognitive Accessibility through a Known AAC

As shown in [6], accessibility and usability improved when using a known communication tool as an interface. Studying this success, the authors found several parallelisms between this AAC and home control interface, which were tried to keep and enhance for all services:

1. Use of pictograms and their sequencing to form sentences in AAC finds parallelism with the way an ordered set of pictograms forms a procedure compound of several tasks to perform. This can be applied to planning a time interval of the class agenda, to the procedure to follow in behavior contention dynamics, or to a script to play in context anticipation.
2. Use of nested and branched navigation structures: TICO uses nested navigation structures among semantic categories to find the concept to express. Home control uses a nested navigation structure among spaces of the house—rooms—among the different elements that can be controlled in a room, and finally among the different functions of each element. Nesting navigation of the agenda can display it in several degrees of detail. The same applies to scripts for context anticipation.
3. Dynamics for advancing through options and selecting one in the interface are the same for TICO and all four services. In fact, TICO can be used as an editor in home control to design interface panels, in time orientation to make the class agenda, in behavior containment to express and show the child the behavior sequence needed to obtain a reward, and finally in context anticipation as a way to edit the scripts.

#### 3.1.3. Cognitive Support in Time Orientation

Personal autonomy is enhanced when children develop skills—or automatisms—to manage time and agenda planning in a somehow normalized way. Time location and time perception are involved in home control for there are tasks to do over the environment that depend on time. Examples may be temporized actions and adapting the house to daylight or temperatures levels as the day progresses. Time perception is also involved in behavior contention and emotion management dynamics, for emotions and impulses have their own evolution that fades in time. The support of the perception of time passing helps children in obtaining emotion and behavior contention. Theater plays scripts in context anticipation also involve time management, both to follow the script sequence and to follow normalized time ranges in social interaction.

In our work, this cognitive area is decomposed into five items. This follows ICF classification of human functions regarding time orientation, and includes goals requested by teachers:

1. time perception, duration of agenda items
2. location in time
3. anticipation of changes and management of anxiety related to them
4. sequencing, dependencies, and order in tasks and events
5. agenda planning, time calculi, time as a resource.

#### 3.1.4. Cognitive Support in Personal Awareness

Home control provides children with an awareness of their environment and their own capacity to interact with it. This is reported by teachers to improve self-esteem, improving the image children have of themselves as their personal awareness.

Personal awareness is also involved in time orientation, as it provides for awareness of location in the time of day and evidences what children can accomplish in a certain time. For most advanced children, the work aims to improve their awareness of time as a resource and their own capacity to do things in a specific time interval.

Behavior contention highlights personal awareness of children’s disruptive tendencies. Context anticipation places children in individual and social interaction situations which rise emotions in them. The training goal is to raise self-awareness of such internal states with the guidance of their teacher, to improve and educate children’s performance. Role playing with different attitudes and tempos in the same situation may widen the spectrum of behavior of a child, increasing his/her own awareness of their internal resources. Context anticipation service offers teachers simple controls to pause, slow down, control sound, and dim or make light brighter.

#### 3.1.5. Social Interaction Support

Generally speaking, groups offer a more elaborated scenario to train children’s autonomy, for social relation arises situations in which children may find conflicts, rush ups, and emotional blockages. Groups also may facilitate tasks performance and learning due to imitation among participants and help in group behavior containment or achieving group goals.

All services are designed for individual and group activity. They do so by implementing individual and multiuser interfaces so that teachers can combine both.

### 3.2. Required Functions per Cognitive Areas and per Service

Table 1 resumes significant functions that each service development is required to fulfill. These functions guided the development of the technological platform and services and determine its technological structure and design, as is detailed at the end of each section.

#### 3.2.1. Services Requirements in Virtual and Conceptual Representations

**Figure 1:**
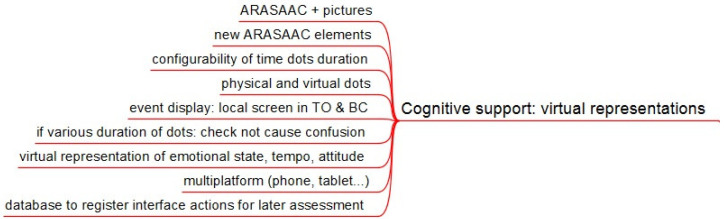
Services requirements for virtual representations.

All four services must adapt their virtual representations to children’s AAC. The pictograms used are the ones in the ARASAAC set. Services need some concepts that currently are not in such set, as time duration, representation of the emotional state, attitude, or tempo. For those, new pictograms will be created. The semantic principles and aesthetics are to be kept as much as possible. Figure 1 summarizes the content of this section.

As there are children whose cognitive level requires pictures instead of pictograms, the services will be able to incorporate any image.

As far as time duration of each element, semantic coherence is compromised with time flexibility. There is some evidence in the literature about an optimum comprehensible unit of 15 min for children in an educational environment. Our previous work with the elderly in shelter homes suggested the use of units of 30 min. The service of behavior contention requires much smaller time intervals and more detailed display of time passing (teachers suggested 1 min). So technology is required to be configurable at this point, and research needs to be done to check whether this semantic difference is acceptable or there is the need to differentiate large time intervals representations from smaller ones.

The elements that originally represented time were physical luminous elements (LEDs) which were suitable for children with low abstract capacity. This forces having some specific non-mainstream hardware. Flat time displays (dots on a screen) are more difficult to understand by some children but improve in materials availability. This work considers both options to provide flexibility to adapt to children’s needs.

Regarding time orientation, teachers requested that the display of the pictogram of the current agenda item should be displayed locally. A small color LCD screen should be added to the time display unit. It will also be used for behavior contention service. 

The large variation regarding the physical and sensory capacities of children to access a screen makes it necessary to develop multiplatform services interfaces, as they need to be displayed and interact on a smartphone, tablet, or computer screen.

In order to have objective data to assess how well children associate virtual and real elements, the platform will keep a register of actions and times over the interface for all services. Access to this register will be protected by standard security measures. This record for assessment is to be considered in the development of all services and for all cognitive areas.

#### 3.2.2. Services Requirements Through a Known AAC

**Figure 2:**
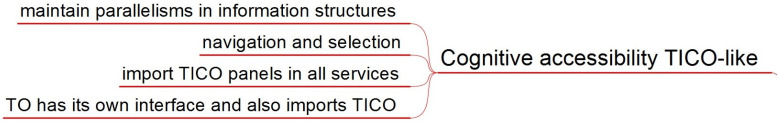
Services requirements for cognitive support through the use of an AAC.

Attention is to be paid to the parallelism described in Section 3.1 regarding semantics, information nested structure, and selection and navigation methods, to take advantage of its cognitive similarity. Figure 2 summarizes the content of this section.

There is a request to use TICO directly to build home control interfaces, agenda plans, behavior procedures, and context anticipation scripts. Once edited with TICO, those interface panels are to be imported into the services. In the time orientation service, configuration needs are too large to be implemented just with TICO, so there will be two ways to program the agenda. One is editing and importing TICO panels with basic information about tasks, sequence, and duration incorporated. The second is directly through a configuration interface that will allow pictogram association with other possible fields as pre-recorded sounds (as their mother’s voice), dot color, and intensity.

#### 3.2.3. Services Requirements in Time Orientation

Time orientation is to be integrated with home control to have agenda items triggering come control actions, and have timer-triggered or temporized actions.

Time orientation service will be able to create a weekly agenda where tasks and events can be included and configured: their sequence, duration, associated images, and sounds. It must also provide notifications to anticipate changes and prepare children to minimize associated anxiety. By default, notifications are given by playing locally pre-recorded sound. Requested remote monitoring and support require messaging through smart phone apps.

Behavior contention and context anticipation will use a time orientation service to represent associated time duration for a task, behavior dynamics, or role play.

Services may need to access agenda information, which suggests the use of centralized registers. This is also required by the multiuser mode of time orientation service. This raises the need for access security management. This database can also be used to register events and uses of the services to provide data for later assessment and research.

**Figure 3:**
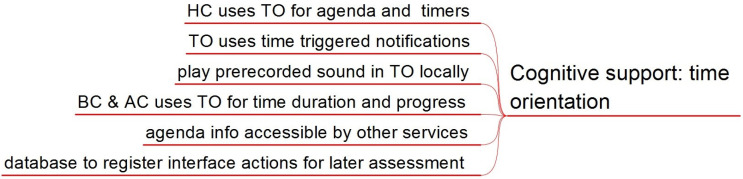
Services requirements for cognitive support in time orientation.

Figure 3 summarizes the content of this section.

#### 3.2.4. Services Requirements in Personal Awareness

**Figure 4:**
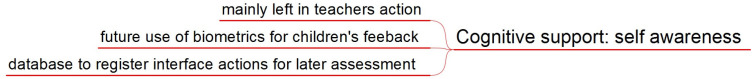
Services requirements for cognitive support in self-awareness.

The scope of this work leaves in the teacher’s hands the support for this cognitive area. Teachers are suggested to signal children about their internal status, being it anxiety, comfort, or emotional well-being among others. This will depend on the child’s capacity regarding introspection, which will be assessed by teachers. Some ideas about direct feedback of the internal activation level of the children (hypoactive—medium—hyperactive) have been considered, as color feedback or notifications when some biometric thresholds are reached, which are left for future work. Figure 4 summarizes the content of this section.

#### 3.2.5. Services Requirements for Social Interaction Support

**Figure 5:**
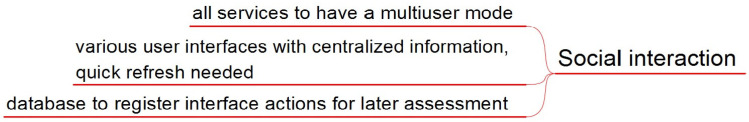
Services requirements for cognitive support in social interaction.

As an educational tool, all services require multiuser mode to support group activities in the class. Frequently, children’s cognitive capacities will be very different from each other, so this leads to the requirement of individualized interfaces operating over the same centralized system or database. A quick enough refresh of the interfaces correlated to the database is needed, for the status of each element in home control to provide for real time perception. Figure 5 summarizes the content of this section.

## 4. Technology Description and Results

Technological development has not been a linear process, for requirements have been evolving through time as the trials with the prototypes were progressing. What is shown here is a final compilation of the current state of the platform and some aspects of the services.

### 4.1. Platform Description

**Figure 6:**
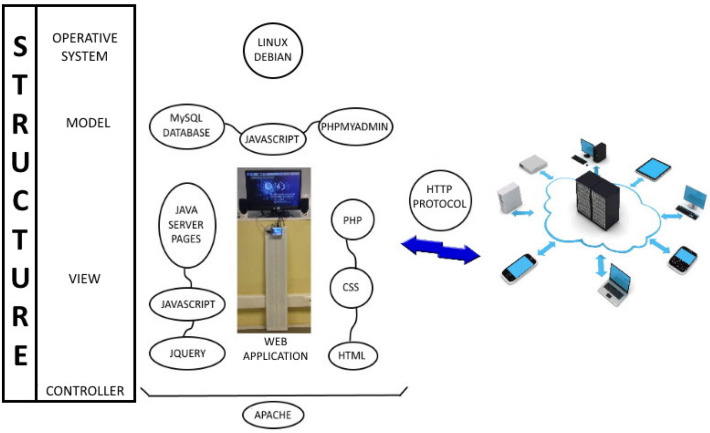
Software elements of the control system of the platform.

The platform is the piece of technology that provides for common functions to all services, which is also used to centralize, register and share their information. The software architecture of our platform is shown in Figure 6, modeling overall relations among the different entities and providing the structure and methodology for software development. All modules have been chosen to make the working environment easier, more comfortable, and usable.

Some of the software modules used are PHP, Ajax, Bash, and Python for hardware interaction; HTML, CSS, PHP Javascript, and JQuery for the human-machine interfaces; and MySQL and phpMyAdmin for databases and table management.

Common functions that are implemented in the platform are:

1. User access and control
2. Platform action control
3. Gather alarms and notifications produced in the platform
4. Save services configurations (communications, power supply faults, panel names…)
5. Query current data (connected users, agenda, alarms, active services, etc.).

**Figure 7:**
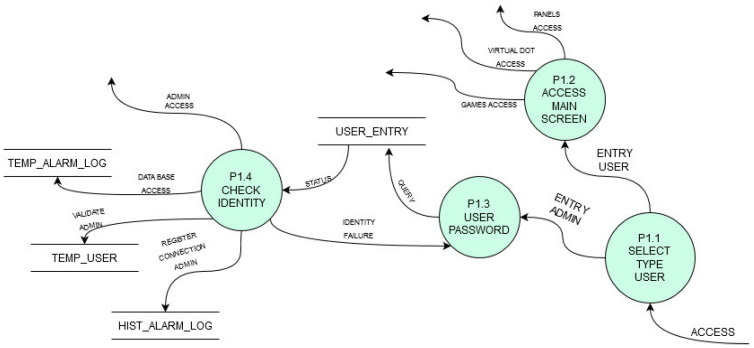
Platform user access control defined in DFD.

**Figure 8:**
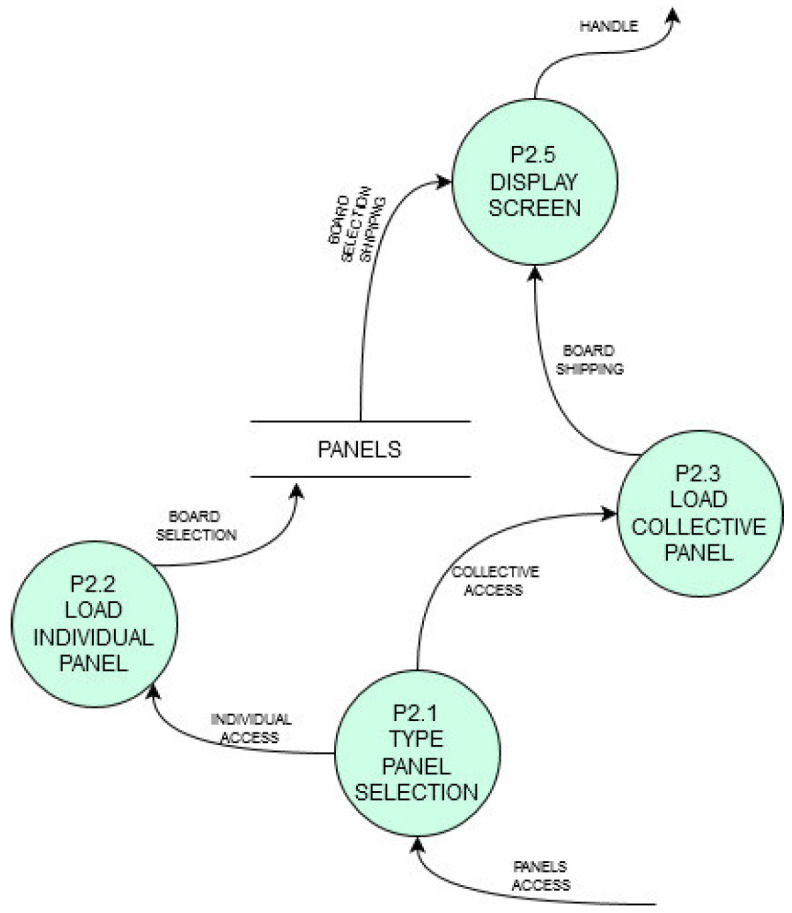
Loading panels process. It may be individual or group panels.

Each function is defined in a Data Flow Diagram (DFD) as shown in Figure 7 to describe the pursued functioning. Figure 7 shows the 2nd level DFD for the process of user access control managed by the platform. Figure 8 shows another platform process (also 2nd level in DFD) which shows the process of loading individual or group panels into the service.

**Figure 9:**
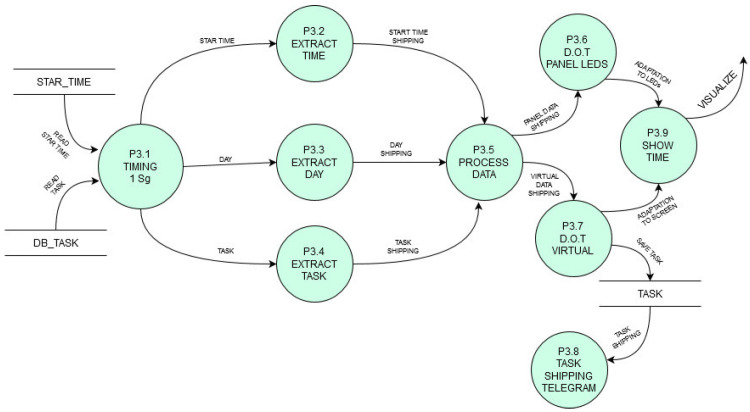
Second level in DFD format for TO service with a virtualized display.

Each service will also have its own processes described in a similar DFD format. Just to illustrate it, the virtual TO service has the data flow functioning shown in Figure 9.

#### 4.1.1. Database Structure

##### Interaction among Services

All services designed so far in the AAL platform register and retrieve internal variables from central databases. This allows several applications to run over the same set of data. On one side, design gets sensible as applications may change the functioning of others dynamically, so data access needs to be looked at in detail, and the authorizations scheme needs to be set. Regulating the rights of each other service to access the central database allows for selective interaction at a functional level.

##### Multiuser Capabilities

Multiuser functioning of services also requires databases as a technological structure. This way, information flow is set to and from each participant’s panel, and group dynamics can be implemented with any of the four services developed.

For multiuser functioning of services, several instances of the same application can be launched simultaneously. This allows having several personalized home control panels or personalized time orientation displays. This favors cognitive accessibility by allowing adaptation of interfaces to each participant: each individual may access and possibly interact with a configurable subset of elements of each service. In time orientation, notifications can be generated only in the configured individual interfaces when they are virtualized.

**Figure 10:**
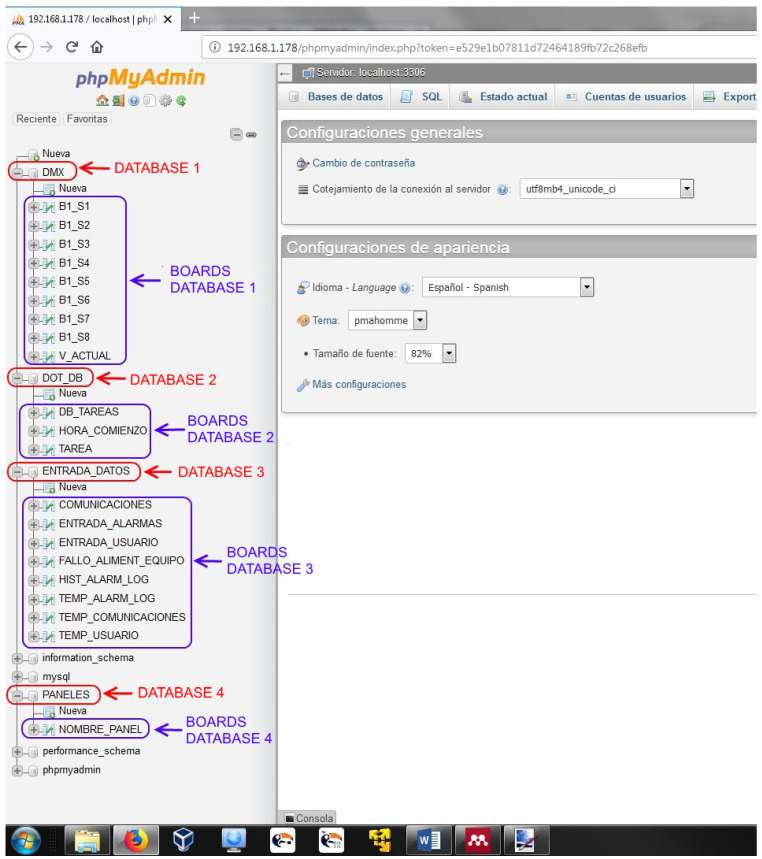
Platform databases.

As shown in Figure 10, databases are compiled in one set and differentiated by the type of information they contain: elements status, time orientation events, data input, home control panels, among others.

#### 4.1.2. Communications

**Figure 11:**
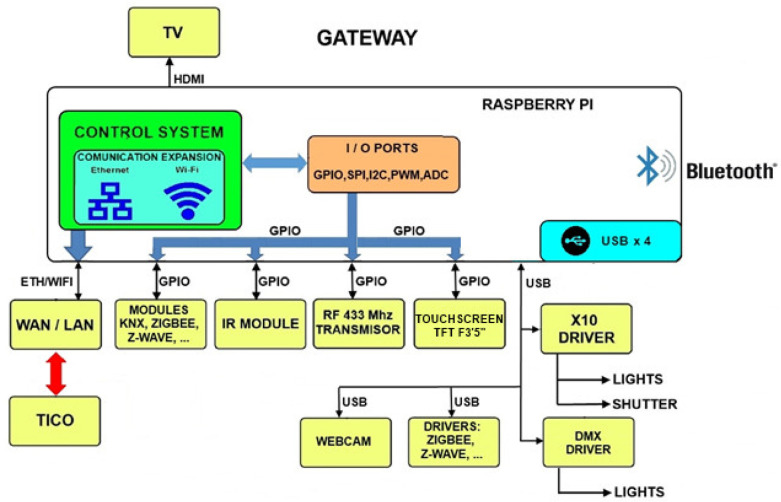
Platform and home control communication elements.

The platform also takes care of communications and has the capacity to enable or disable them. Access can be done by a local network (through IP) or from an external one using a DDNS service. It also has the possibility to use the Telegram app as a message service to send the current programmed task to a teacher, relative, or caregiver. A modular diagram is shown in Figure 11.

**Figure 12:**
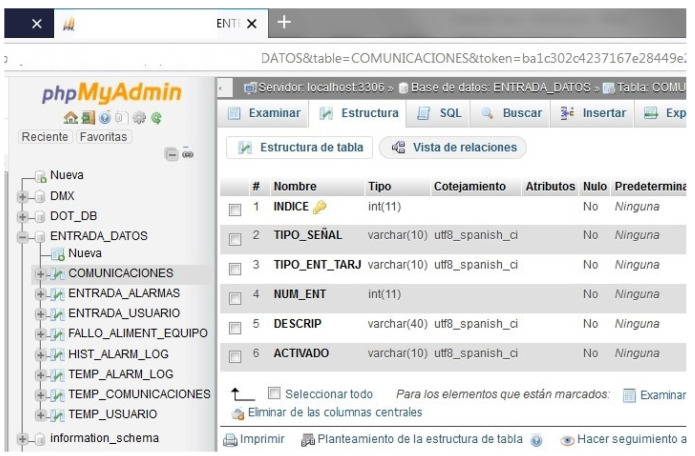
Platform communication table structure.

Several operative information is stored in the form of tables, with which one can enable and disable platform services as communications and change any other parameter. Figure 12 shows the table structure used for this platform communications function.

Some other examples of tables used are:

- platform alarm enabling and user alarm enabling
- user control enabling
- power supply faults and historical register of actions
- active communication services
- TO programmed tasks
- other stored elements: panels, DMX elements (DMX is a communication standard for theater and scenery arts technology).

### 4.2. Platform Adaptation to COVID-19 Situation of Confinement

Due to the sanitary alarm situation derived from COVID-19 arriving in Spain, children were confined in their homes, changing their circumstances completely and forcing a fragile cognitive population into tele-education and tele-support dynamics. To put these services available at children’s homes, the platform was migrated from its single-board computer hardware (Raspberry PI) to a Linux server running from the university dependencies. All specific hardware was not available, so only virtualized elements and time agenda were left. Through any videoconferencing system, the teachers could communicate with students via TICO and could interact with them with those services by screen sharing.

### 4.3. Home Control Service Description

Home control was the first service to be built with TICO as a user interface. TICO uses a hierarchical structure to navigate through semantic categories: p.e., foods/first plates/vegetables, and so on until the needed idea is found (its word/picture/pictogram…). So it has the capacity to configure which new page to go when a pictogram is selected. Once selected it can be placed in a panel to become an element of the sentence being built. Each element has an associated sound to allow the reading of the sentence. So it can build ideas through the association of terms and have them read later in the language chosen.

Home control uses those built panels “the sentences” as areas in the house, i.e., rooms. Navigation through rooms and then through elements in each room that are available for control. When selecting an operation over a final element an order is sent to the hardware device through the platform. Communication service translates it into the device data protocol to perform the required action, as turning on a light or increasing the volume of a TV set.

It admits pictures and pictograms, and the amount and position of the elements are edited as a communication panel. Panels can be individualized to different screen sizes, space between elements to facilitate selection for coarser or finer psychomotricity, and limit the number of items or navigation levels to adapt to different cognitive capacities. This also allows adaptation to different scenarios, such as school or classroom, home scenarios, or other institutional scenarios as shelter homes or day centers.

Home control service has been installed at Alborada’s library for more than two years now. Results are very positive, as detailed in [6].

**Figure 13:**
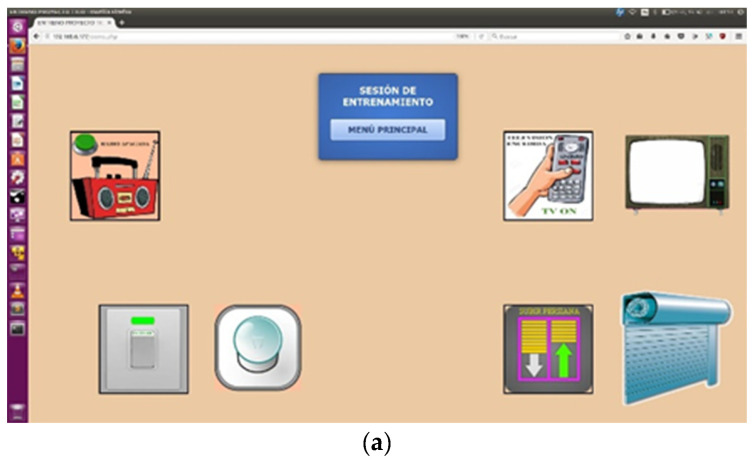

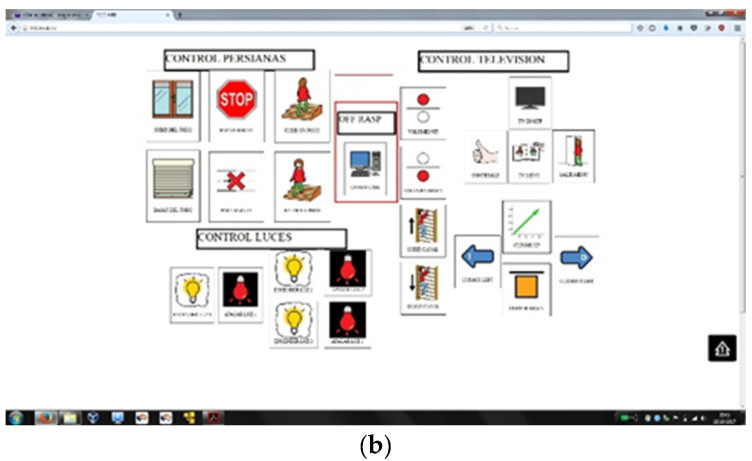
TICO–home-control: interface accepts pictograms, drawings, or pictures to adapt to several cognitive accessibility conditions: (**a**) interface for training at the school library; (**b**) interface for a room.

This work learns from insight into the possible mechanisms of this success and extrapolates them into the other services, as explained in the first sections of this article. Flexibility in interface design was greatly appreciated by teachers. Direct effects in the real world were found very interesting for cause-effect training in children with lower cognitive skills. The system is ready to be installed at homes. Figure 13 shows different versions of its interface, (a) is for training at the school library and (b) shows a prototype for a room.

### 4.4. Time Orientation Service Description

**Figure 14:**
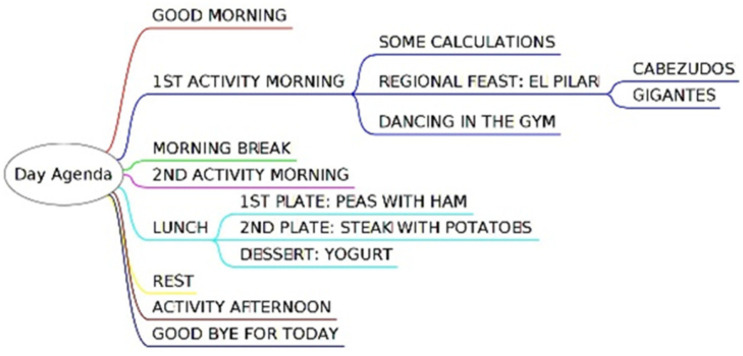
TICO’s hierarchical structure in time orientation allows grading level of detail in agenda.

The display in time orientation service can be as simple as a row of 5 dots -meaning there are 5 min to wait to start the desired video playing- or as complex as a full-day agenda for the class giving additional details for the individual activities, some pupils will need to attend, as logotherapy, physiotherapy or gym. TICO editor easily supports such setups. Hierarchical and spatial information here has a different meaning than in-home control: spatial sequence will reflect time sequence, and hierarchy is used to offer different levels of detail when addressing a time interval. As Figure 14 shows, hierarchy allows us to keep a low number of items per screen and have navigation through them in increasing detail level: One can have a simplified day agenda with only six items: the first part of the morning activity, the break in the middle of the morning, the second part of the morning, time to eat and rest, continuation in the afternoon and finally going home. When selecting the time to eat and rest, the user gets details as the plates in the menu or any other hierarchical level considered appropriate for the child or for the class.

Spatial distribution of elements will mean sequential progress in time, so the position of elements in the phrase means their time location. Some specific elements were added to express time duration and moment in time coherently as it is done in other temporal orientation assistive tools.

Cognitive accessibility makes it necessary to be able to display large moments in time through images (rising, morning, midday, afternoon, sunset, night) or give an increasing number of details for the most capable children ending in analog clocks drawing or numbers.

**Figure 15:**
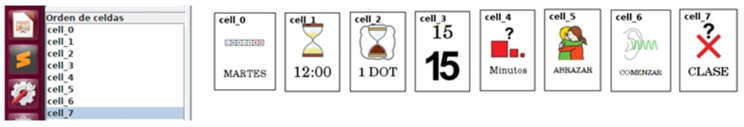
Simple ways to express duration.

Cells 0 and 1 in Figure 15 are introduced for time location. Cells 2, 3, and 4 show different styles of expressing time duration for higher cognitive levels. In classes where the cognitive level is lower, a dot will keep its standard duration, 15 min, to avoid confusion until further research is done to assess how to display different time scales. Technology is prepared to configure the duration of each dot.

**Figure 16:**
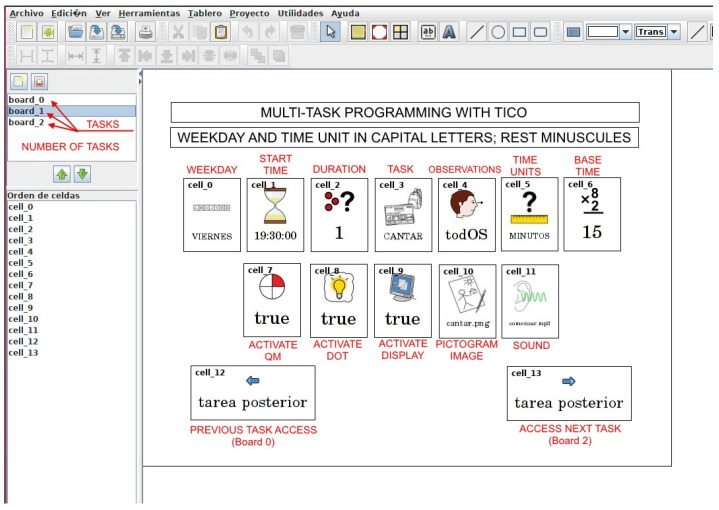
Elaboration of class agenda with TICO for later import from TO service.

**Figure 17:**
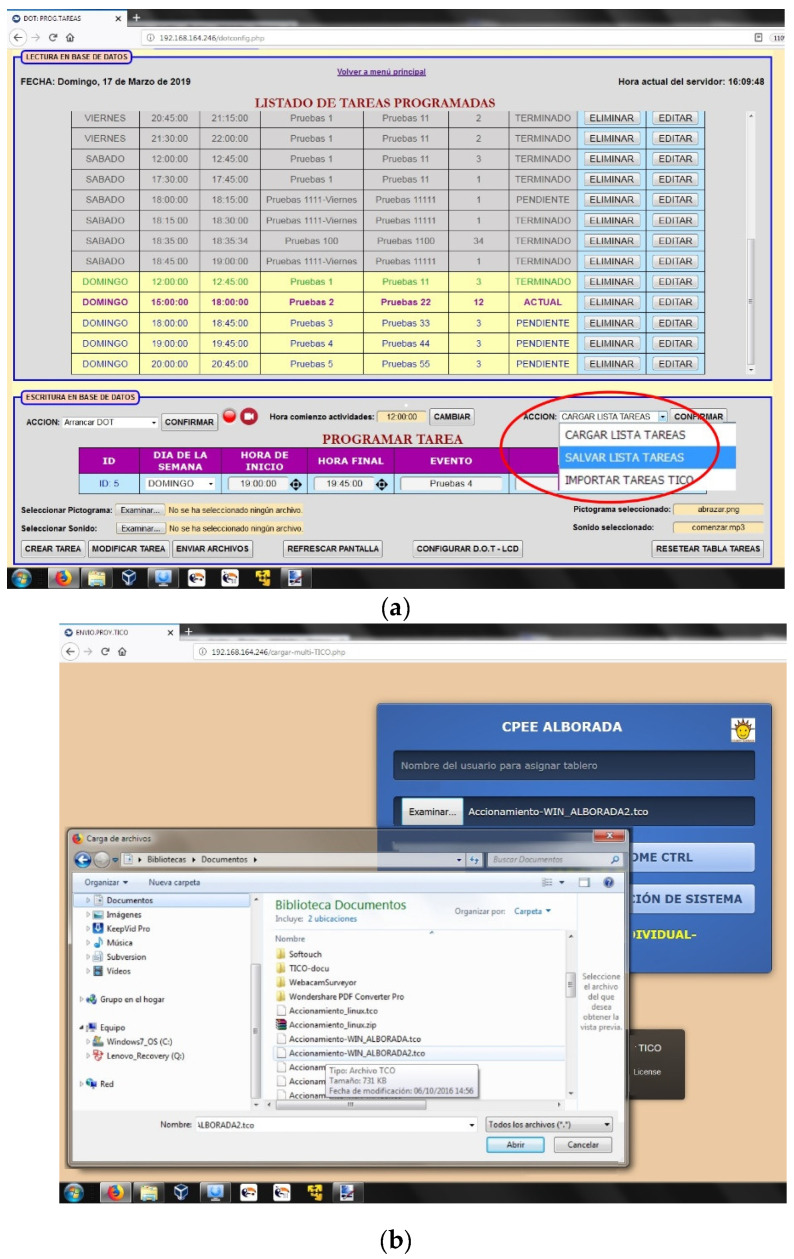
(**a**) Import from TICO in time orientation service; (**b**) TICO file selection.

Children may edit their own time interval agenda with TICO. Figure 16 shows an example of agenda editing in the TICO panel. Those panels will be exported to the time orientation service and display panel and followed by the lighting pattern of the row of luminous elements as shown in Figure 17a,b.

**Figure 18:**
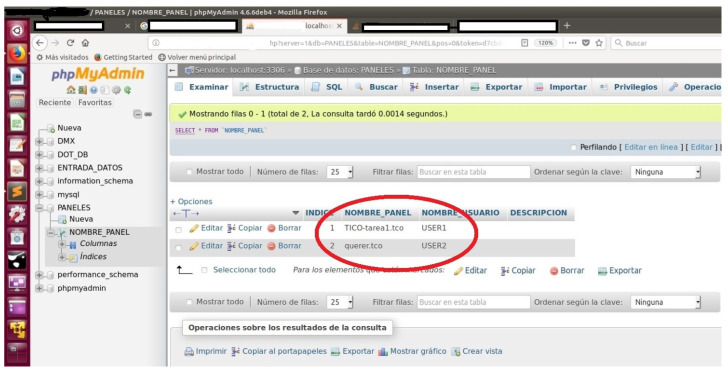
View of the database where TICO panels are imported into time orientation service and associated to different users.

This information is then registered in a mysql database, so other services in the platform with enough rights can interact with the time orientation service, both for reading and writing. Figure 18 shows how two TICO panels have been imported into the time orientation service for user1 and user2. “tco” extension is the one TICO uses.

The current scheme of TO service distinguishes between two types of users, teachers who configure the tool and children who receive the information and may do exercises in making up the sequences and duration of the agenda—which is only one part of service configuration.

Testing of the impact this service had over the personal autonomy of children validated the previous version of this service as far as personal autonomy improvement. Those trials were based on IFC items, and also showed subjective improvement in following class agenda and decrease of anxiety associated with changes. Yet, it was not clear if children had improved their skills and understanding or just had developed habits and automatisms. 

Further trials with the KaTiD evaluation methodology, which specifically assesses the understanding and skills in time management, were a surprise for teachers. The cognitive level required for those tests was far away from children’s capacity. Then teachers realized those children could show good performance without having acquired understanding, so it was concluded children had developed automatisms. This showed up a need for tests with lower capacity levels and also the need for training.

Time orientation service has experienced a significant conceptual and operative improvement with the addition of cognition accessibility and training dynamics. The incorporation of edition panels and training activities with them are expected to work as they did with the Home Control service. The goal is to establish the conceptual parallelisms that allow children to improve their time understanding and management. TO’s feature of importing TICO’s panels supports this training with a class of children, so they can make their agenda with TICO both in individual and group mode, taking advantage of imitation and previous cognitive integration through their AAC.

The other services will also benefit from the development of TO, both for time orientation skills improvement and for agenda information sharing.

An education research assessment is pending to further validate its underlying cognitive mechanisms.

This service was also studied for other target populations and scenarios. It was installed at the homes of people with mild dementia, and also studied for a shelter home and day-center as a time orientation tool. Migration to that other population target is expected to show benefits in senior life quality, especially in early or mild stages of dementia or other cognitive difficulties. Research is easier with children for they show stable capacities during long periods of time, while the quick deterioration of cognition of elderly people with dementia may hide potential benefits from this approach.

### 4.5. Behavior Contention Service Description

Behavior contention service, from a technological point of view, can be understood as a particular case of time orientation service focused on a small time interval. It needs to show one sequence of events, instead of an all day agenda, and the time progress display is also reduced to a specific time interval in which contention needs to be achieved. Time perception support must be much more detailed, in the order of a minute or less, to facilitate the adherence of attention of the child to the time progressing display and derive his/her attention from the impulses that produce disruptive behavior.

Behavior contention service came “directly from the field”. It was tried by the teachers to tackle difficult situations that were repeated day after day.

Its evaluation has been so far conducted freely by teachers on a fully user oriented basis, to solve each user case that came up, raising evidence of its usefulness.

**Figure 19:**
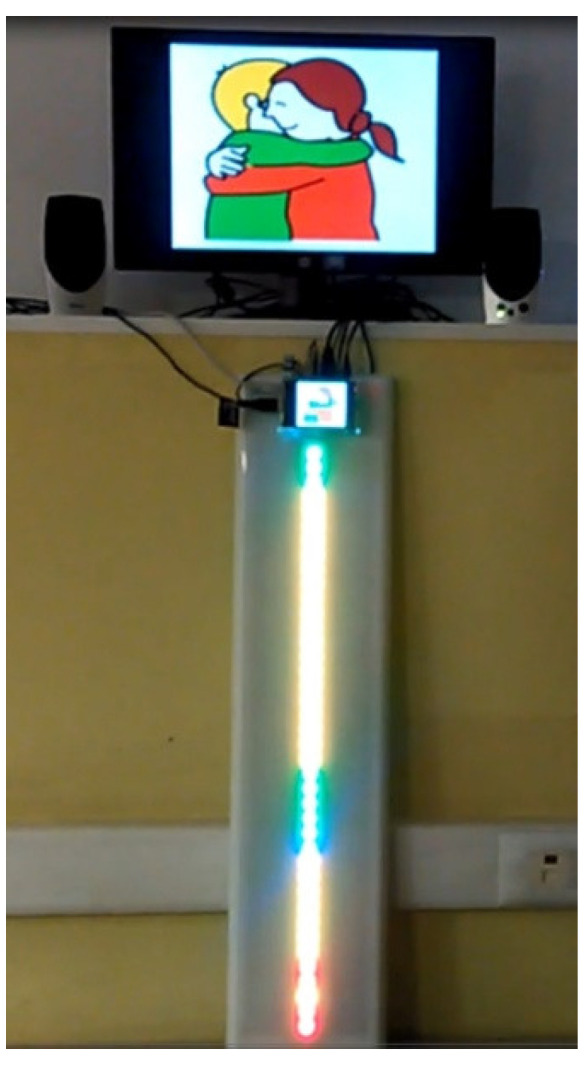
Reward achieved in behavior contention service.

When starting, the screen shows the behavior sequence to perform as a condition for the reward to be achieved. Figure 19 shows an example of reward after contention: the screen is showing the pictogram representing the reward. So far, the coherence of representation of duration is not yet solved. Teachers use the same physical element to represent 15 min for a class agenda and to represent one minute for behavior contention. As it seems so far, the potential confusion is not shading the good outcome of training behavior contention.

### 4.6. Context Anticipation Service and Sensory Room Description

Context anticipation was originated as a multiuser evolution of the sensory stimulation room. It uses its elements transferred to a class to be used by a group of children and takes special advantage of multimedia immersive capacities.

**Figure 20:**
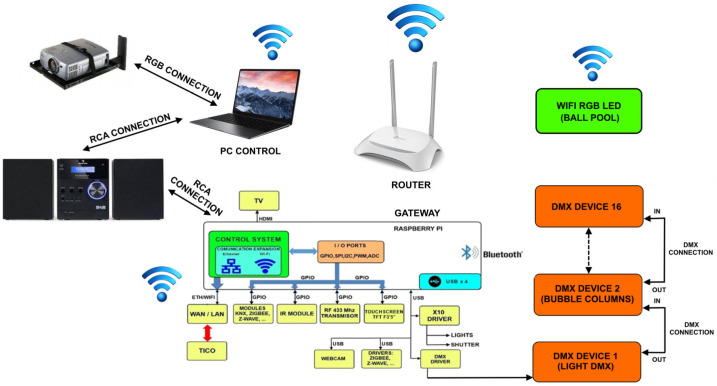
Communication scheme for context anticipation service.

The script is worked in TICO format. Technology, though, follows the same scheme of sensory stimulation room, which is shown in Figure 20, very similar to the home control system. Specific elements are added, as video projectors and DMX communication protocol to integrate devices from the scenic arts field as lights, odor spraying, vibration, and sounds. DMX communication protocol is the standard used in theater and other scenic arts. As far as communications, it uses the platform communication facilities, so it becomes a powerful tool to integrate almost any available device with no proprietary protocol.

**Figure 21:**
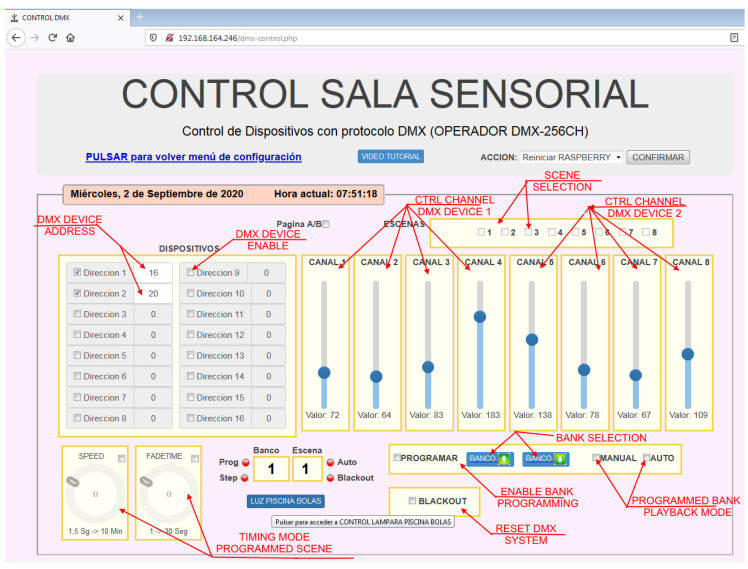
Teacher’s interface for control in context anticipation service.

Interface for technology operation offers teachers full control of each element, resembling a DMX control panel as shown in Figure 21. This panel can be displayed and interacted with on a smartphone, computer, or tablet.

The context anticipation service was installed at school right before the COVID-19 pandemic, so it has been tested by teachers but not with children.

**Figure 22:**
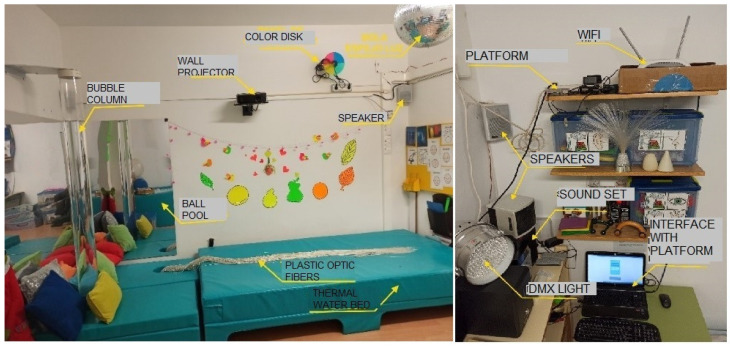
Preceding system of context anticipation: Alborada’s sensory stimulation room.

Its elements are installed and working in the sensory stimulation room at school, as shown in Figure 22. Sensory room elements are connected to the same platform, and they can be controlled from several devices like smartphones, computers, or tablets. Its structure, as far as technology is concerned, resembles very much that of home control. The interfaces are different, for context anticipation service focuses on how the environment affects children instead of focusing on children’s capacity to interact with it. Children would get benefit from this work in the near future.

## 5. Conclusions and Future Work

A set of services with enhanced cognitive support is developed inside a platform that provides for common structural functions and interoperability. Platform and services development integrate common support cognitive mechanisms. They are at the same time support and training tools in cognitive and social areas.

The installation has been done at school for all four services, which were tested as far as technological functioning and teachers handling with optimum results.

Home control and time orientation have gone through systematic evaluation processes that evidence their enhanced accessibility and usefulness for support and training purposes.

Teachers have tested behavior contention service in a non-structured way. This service was a success from the very beginning, raising new requirements as they used it. There is the need to check if using different timing for time passing in class agendas and in this service creates confusion due to the semantic difference implied.

Context anticipation service has not been tested with children yet, due to the sanitary crisis in early 2019 that placed a pause in all research activities inside the special education school. Teachers gave positive feedback about its functioning after technology installation.

As future work, it is time for technology developers to give a step back and let education professionals and education research teams take the lead as far as evaluation and further requirements to fulfill. All services allow further experiences for deeper educational and ergonomic research.

There is also work to be done to prepare educational materials based on the resources those services offer, as storytelling with home control, group agenda planning and follow-up, and various scripts and video materials for context anticipation.

As technological next steps, the authors consider non-intrusive biometric measures such as accelerometers to offer personal awareness feedback to children. New requirements for technology are expected regarding context anticipation when experiences with children are finally performed, specifically regarding the introduction of elements from the sensory room and multimedia control for mood and tempo modulation in group settings.

Cognitive and social support looks efficient in service performance and also a way to improve personal autonomy.

## Figures and Tables

**Table 1:** Functions to implement per service to attend cognitive items.

Cognitive Functions to Attend per Service	Virtual Representation	Interface as AAC	Time Orientation	Self Awareness	Social Interaction
**Home Control HC**	Keep coherence and parallelism among virtual and real worlds. Multiplatform to adapt to various sizes.	HC interface is analogous to AAC in access, navigation and semantics.	HC uses time agenda items and temporized scenes.	HC will support context awareness and child’s capacity to interact with it.	HC will implement multiuser functions. Individual interfaces acting on the same HC system are required.
**Time Orientation TO**	Need local display of event. Keep coherence. Include time representation elements. Check acceptance of varied meaning of a dot (15 mins).	TO interface uses AAC to represent tasks and spaces. Agenda editing with TICO and specific interface.	TO uses class agenda format and provides supports for sequence, time location, duration and changes anticipation.	TO will support self-awareness of personal time performance. It also will bring attention of children to their own wellbeing to avoid stress.	TO will provide for group work in agenda planning and executing as a tool for social interaction training.
**Behavior Contention BC**	Need local display of action. Check acceptance of varied meaning of a dot (1 min).	BC interface uses both semantics and sequencing of AAC for the behavior required and TO for time left.	Visualization of time passing and time left uses TO format.	BC will support self-regulation and self-knowledge.	BC will support exercises to reconduct behaviors which influence in child’s social interaction.
**Context Anticipation CA**	Virtual immersive multimedia, new pictograms for emotions, attitudes, tempo.	Script of CA is written with AAC.	Times of role playing are included in CA script. Rhythm can be also indicated.	CA will propose new behavior patterns to widen children comfort zone and resources.	CA will support exercises to train social interaction.

## Data Availability

Not applicable.
